# ﻿*Cladosporium* spp. (Cladosporiaceae) isolated from *Eucommiaulmoides* in China

**DOI:** 10.3897/mycokeys.91.87841

**Published:** 2022-07-26

**Authors:** Si-Yao Wang, Yong Wang, Yan Li

**Affiliations:** 1 Key Laboratory of Plant Resources Conservation and Germplasm Innovation in Mountainous Region (Ministry of Education), Guiyang 550025, Guizhou Province, China; 2 College of Life Sciences/Institute of Agro-Bioengineering, Guizhou University, Guiyang 550025, Guizhou Province, China; 3 Department of Plant Pathology, Agriculture College, Guizhou University, Guiyang, Guizhou Province, 550025, China

**Keywords:** Asexual morphs, new species, phylogeny, taxonomy

## Abstract

*Eucommiaulmoides* is a rare tree species in China with high medicinal and gum value. Nine strains of hyphomycetous fungi were isolated from the leaf litter of *E.ulmoides* in Guizhou Province. Preliminary identifications based on ITS indicated that they belong to the genus *Cladosporium*. Morphology and phylogenetic analyses based on the internal transcribed spacer regions (ITS) of the nrDNA, the partial translation elongation factor 1-α (*tef1*) gene and partial of actin (*act*) gene confirmed that the strains represent four species, including two novel taxa, viz., *Cladosporiumeucommiae* and *C.guizhouense* and two new substrate records for known species.

## ﻿Introduction

*Eucommiaulmoides* Oliver (‘du-zhong’ in Chinese), the single extant species of *Eucommiaceae* (related to *Ulmaceae*), is a dioecious, wind-pollinated tree evenly distributed in mixed mesophytic forest habitats of valleys, hills, and low mountains in central and eastern China (Cronquist 1981; Zhang 2016). *E.ulmoides* is widely cultivated in China and other countries owing to its high medicinal and gum value.

The fungal genus *Cladosporium* was established by Link (1816). Cladosporium (Cladosporiaceae) is a ubiquitous genus in *Dothideomycetes* (Abdollahzadeh et al. 2020). This genus is widely distributed throughout the world and isolated from various sources such as air, soil, plants, food, debris, cloth, paint and other organic materials (Ellis 1977; Bensch et al. 2010, 2012, 2018; Temperini et al. 2018; Chung et al. 2019). Most *Cladosporium* species are saprobic (Bensch et al. 2010), and they occur on various senescing and dead leaves and stems of herbaceous and woody plants (Brown et al. 1998; El-Morsy 2000). The morphology of *Cladosporium* is mainly characterized by its asexual morph, which comprises differentiated conidiophores producing acropetal chains of conidia from mono- or polyblastic conidiogenous cells (Isabel et al. 2021). Both the conidiogenous cells and conidia show conidiogenous loci (scars) with a distinctive coronal structure, which is composed of a central convex dome surrounded by a raised periphery, usually thickened, refractive and dark (David 1997; Isabel et al. 2021). A molecular approach combined with morphological features has recognized more than 230 species in *Cladosporium*, which are grouped into three species complexes, i.e., the *C.cladosporioides*, *C.herbarum* and *C.sphaerospermum* complex (Schubert et al. 2007; Bensch et al. 2010, 2012, 2015, 2018; Sandoval-Denis et al. 2016; Marin-Felix et al. 2017).

In a recent research program, we have carried out a survey of micro-fungi associated with *E.ulmoides* in a forest in China. In this study, four *Cladosporium* taxa were isolated from fallen leaves of this plant species in Guizhou Province, including two new species, namely *C.eucommiae* and *C.guizhouense* spp. nov., which are introduced based on morphology and phylogenetic analyses. Newly generated molecular data, descriptions and illustrations of *C.tenuissimum* and *C.perangustum* are also provided herein.

## ﻿Materials and methods

### ﻿Sample collection and fungal strains isolation

Fallen leaves of *E.ulmoides* were collected in a forest plantation of Guizhou University, Guiyang, Guizhou Province, China, in January 2021. The samples were stored in envelopes and several topsoil samples from the forest were stored in self-sealing bags, then taken back to the laboratory and photographed. Before isolation, collected leaves samples were sprayed two to three times with 75% ethanol to disinfect the leaf surface. Pure cultures of the fungi were obtained by single spore isolation (Chomnunti et al. 2014). Fungi in the soil samples were isolated by the dilution plate method (Zhang et al. 2015). A small amount of soil (1 g) per sample was collected and added to 9 mL of sterile water in a 15 mL sterile glass test tube. It was manually mixed and then the suspension was diluted to a series of concentrations (10^–1^, 10^–2^, 10^–3^, 10^–4^, 10^–5^ and 10^–6^), and 100 µL from each concentration was spread onto 90-mm-diam Petri dishes containing Synthesis of low nutrient Agar (SNA), Potato Dextrose Agar (PDA), Malt Extract Agar (MEA) and Oatmeal Agar (OA) (Zhang et al. 2017). These SNA, PDA, MEA and OA plates were incubated at constant temperature (25 °C) in a controlled temperature light incubator. Holotype specimens of the new species were conserved in the Herbarium of the Department of Plant Pathology, Agricultural College, Guizhou University (**HGUP**). Ex-type cultures were conserved in the Culture Collection at the Department of Plant Pathology, Agriculture College, Guizhou University, P.R. China (**GUCC**).

### ﻿Morphological description

Pure cultures were grown on SNA, PDA, MEA and OA media in a constant temperature incubator (25 °C). Culture characteristics were recorded and examined using a dissecting microscope (LEICA S9i, Germany). The morphological observations and measurements on SNA were made using a Zeiss Scope 5 compound microscope (Axioscope 5, China) with an attached camera AxioCam 208 color (ZEN 3.0) and measurements were made using ZEN 3.0. Taxonomic information for the two new taxa were deposited in MycoBank (www.mycobank.org).

### ﻿DNA extraction, PCR amplification and sequencing

Fresh mycelia were scraped from the PDA plates with a sterilized scalpel. Genomic DNA was extracted using Fungal gDNA Kit (Biomiga #GD2416, San Diego, California, USA) in accordance with the manufacturer’s instructions. PCR amplification was performed in a 25 μL reaction volume following Liang et al. (2018). Primer pairs ITS4/ITS5 (White et al. 1990), EF1-728F/EF1-986R (Carbone and Kohn 1999) and ACT-512F/ACT-783R (Carbone and Kohn 1999) were used for ITS, *tef1* and *act*, respectively. The amplification procedures were performed using the method described by Halo et al. (2019). Purification and sequencing of these three gene loci were carried out by the SinoGenoMax company (Beijing, China).

### ﻿Phylogeny

Sequences used in this study (Table 1) were assembled based on the closest matches from the BLASTn search results (https://blast.ncbi.nlm.nih.gov/Blast.cgi) and previous publications (Sandoval-Denis et al. 2016; Bensch et al. 2018; Halo et al. 2019). Alignments were conducted with the online version of MAFFT v. 7.307 (Katoh and Standley 2016), checked visually and improved manually where necessary using BioEdit v. 7.1.3.0 (Hall 1999). SequenceMatrix v. 1.7.8 (Vaidya et al. 2011) was used to concatenate the aligned sequences of the different loci. Ambiguous areas were excluded from the analysis using AliView (Larsson 2014) and gaps were coded as missing data.

**Table 1.** Taxa used for molecular phylogenetic analyses and their GenBank accession numbers. Newly generated sequences are in bold. (^T^) = ex-holotype strain, (^ET^) = ex-epitype strain, (^NT^) = ex-neotype strain.

Species	Strain number	Host	Country	GenBank Accession number		
				*ITS*	*tef1*	*act*
*Cladosporiumangulosum*	CBS 140692^T^	Man, bronchoalveolar lavage fluid	USA	LN834425	LN834521	LN834609
*C.angulosum*	CPC 11526	Acacia mangium	Thailand	HM148127	HM148371	HM148616
*C.anthropophilum*	CBS 140685^T^	Man, bronchoalveolar lavage fluid	USA	LN834437	LN834533	LN834621
*C.anthropophilum*	CBS 117483	–	USA	HM148007	HM148248	HM148494
*C.anthropophilum*	CPC 22272	Indoor air sample, ship	USA	MF574171	MF574173	MF574175
*C.cladosporioides*	CBS 101367	Soil	Brazil	HM148002	HM148243	HM148489
*C.cladosporioides*	CBS 112388	Air, indoor environment	Germany	HM148003	HM148244	HM148490
*C.cladosporioides*	CBS 113738	*Grape bud*	USA	HM148004	HM148245	HM148491
*C.colocasiae*	CBS 386.64T	*Colocasiaesculenta*	Taiwan	HM148067	HM148310	HM148555
*C.colocasiae*	CBS 119542	*Colocasiaesculenta*	Japan	HM148066	HM148309	HM148554
***C.eucommiae* sp. nov.**	**GUCC 401.1^T^**	**Fallen leaves o*f Eucommiaulmoides***	**China**	**OL587465**	**OL504966**	**OL519775**
***C.eucommiae* sp. nov.**	**GUCC 401.9**	**Fallen leaves of Eucommiaulmoides**	**China**	**ON334729**	–	**ON383337**
***C.guizhouense* sp. nov.**	**GUCC 401.7^T^**	**Fallen leaves of *Eucommiaulmoides***	**China**	**OL579741**	**OL504965**	**OL519780**
***C.guizhouense* sp. nov.**	**GUCC 401.8**	**Fallen leaves of *Eucommiaulmoides***	**China**	**ON334728**	**ON383470**	**ON383338**
*C.magnoliigena*	MFLUCC 18-1559^T^	*Magnoliagrandiflora*	China	MK347813	MK340864	–
*C.magnoliigena*	MFLUCC 18-1557	*Magnoliagrandiflora*	China	MK347811	MK340862	–
*C.oxysporum*	CBS 125991^T^	Soil, near the terracotta army	China	HM148118	HM148362	HM148607
*C.oxysporum*	CBS 126351	Indoor air	Venezuela	HM148119	HM148363	HM148608
***C.perangustum***	**GUCC 401.6**	**Fallen leaves of *Eucommiaulmoides***	**China**	**OL579742**	**OL581726**	**OL519779**
*C.perangustum*	CBS 125996^T^	*Cussonia* sp.	South Africa	HM148121	HM148365	HM148610
*C.perangustum*	CPC 12216	*Morusrubra*	Germany	HM148135	HM148379	HM148624
*C.perangustum*	CPC 14247	*Magnolia* sp.	USA	HM148145	HM148389	HM148634
*C.perangustum*	CPC 13870	*Teratosphaeriafibrillosa*	South Africa	HM148142	HM148386	HM148631
*C.perangustum*	DTO 323-E4	Indoor air	China	MF473180	MF473602	MF474028
*C.perangustum*	CPC 22297	Indoor air sample	USA	MF473172	MF473595	MF474020
*C.rectoides*	CBS 125994^T^	*Vitisflexuosa*	South Korea	HM148193	HM148438	HM148683
***C.tenuissimum***	**GUCC 401.2**	**Fallen leaves of *Eucommiaulmoides***	**China**	**OL579746**	**OL504967**	**OL519776**
***C.tenuissimum***	**GUCC 401.3**	**Fallen leaves of *Eucommiaulmoides***	**China**	**OL579745**	**OL505077**	–
***C.tenuissimum***	**GUCC 401.4**	**Fallen leaves of *Eucommiaulmoides***	**China**	**OL579744**	**OL581724**	**OL519777**
***C.tenuissimum***	**GUCC 401.5**	**Fallen leaves of *Eucommiaulmoides***	**China**	**OL579743**	**OL581725**	**OL519778**
*C.tenuissimum*	CBS 125995^E^T	*Lagerstroemia* sp.	USA	HM148197	HM148442	HM148687
*C.tenuissimum*	CPC 12795	*Musa* sp.	Polynesia	HM148209	HM148454	HM148699
*C.tenuissimum*	CBS 126359	*Musa* sp.	USA	HM148198	HM148443	HM148688
*C.tenuissimum*	CPC 10882	*Gnaphaliumaffine*	South Korea	HM148204	HM148449	HM148694
*C.tenuissimum*	CPC 10538	*Musa* sp.	Mozambique	HM148202	HM148447	HM148692
*C.tenuissimum*	DTO 323-C5	Indoor air	China	MF473289	MF473712	MF474139
*C.tenuissimum*	CPC 13252	Rock	Australia	HM148216	HM148461	HM148706
*C.tenuissimum*	CPC 13732	*Shoreasiamensis*	Laos	HM148217	HM148462	HM148707
*C.tenuissimum*	CPC 14196	*Basellaalba*, leaves	Laos	HM148218	HM148463	HM148708
*C.xanthochromaticum*	CPC 11609^T^	Man, bronchoalveolar lavage fluid	USA	EF679356	EF679431	EF679508
*C.xanthochromaticum*	CBS 126364	*Erythrophleumchlorostachys*	Australia	HM148122	HM148366	HM148611
*C.xylophilum*	CBS 125997^T^	*Piceaabies*, dead wood	Russia	HM148230	HM148476	HM148721
*C.langeronii*	CBS 189.54^T^	Man, mycosis	Brazil	DQ780379	JN906990	EF101357
*C.neolangeronii*	CBS 797.97^T^	Indoor environment	Netherlands	MF473143	MF473576	MF473992

The Maximum Likelihood (ML) analyses were carried out at the CIPRES web portal (Miller et al. 2010) using RAxML (Stamatakis 2006). The tree search included 1,000 non-parametric bootstrap replicates and the best scoring tree was selected from suboptimal trees under the GTRGAMMA substitution model. The resulting replicates were plotted on to the best scoring tree obtained previously. Non-parametric bootstrap analysis was implemented with 1,000 duplicates. Maximum Parsimony (MP) analyses were performed with PAUP v. 4.0a (Swofford 2003), using the heuristic search option with 1,000 random sequence addition replicates and tree bisection-reconnection (TBR) with reconnection limit (=8) as the branch swapping algorithm. Maxtrees was set at 5,000. Branches collapsed (creating polytomies) if maximum branch length is zero. The Tree Length (TL), Consistency Indices (CI), Retention Indices (RI), Rescaled Consistency Indices (RC) and Homoplasy Index (HI) were calculated for each tree generated. Bayesian Inference (BI) analyses were performed in MrBayes v. 3.2.7a (Ronquist et al. 2012). Six Markov chain Monte Carlo runs were started, and the random start trees were calculated for 50,000,000 generations and sampled every 1,000 generations. 25% of the trees initially produced were discarded as burn-in. ML bootstrap support (MLBS) and MP bootstrap support (PBS) equal or greater than 70% (Hillis and Bull 1993) and Bayesian posterior probabilities (PP) equal or greater than 0.95 (Hespanhol et al. 2019) are displayed on the edited phylogenetic tree. The phylogenetic tree was drawn with FigTree v. 1.4.4 (Rambaut 2009).

### ﻿Genealogical Phylogenetic Species Recognition (GCPSR) analysis

Morphological and phylogenetically related species were analyzed using the genealogical consistency phylogenetic species identification (GCPSR) model as described by Taylor et al. (2000) by pin-pair homogeneity index test (PHI) (Bruen et al. 2006). The PHI tests were performed in SplitsTree v. 4.17.1 (Huson 1998; Huson and Bryant 2006) as described by Quaedvlieg et al. (2014) to determine the level of recombination within phylogenetically closely related species. The results can be visualized by constructing a split graph using LogDet conversion and the Splits options. The hypothesis of this analysis is if the PHI value is below 0.05 (Фw < 0.05), there is significant evidence for the presence of recombination.

## ﻿Results

### ﻿Phylogenetic analysis

**Figure 1.**
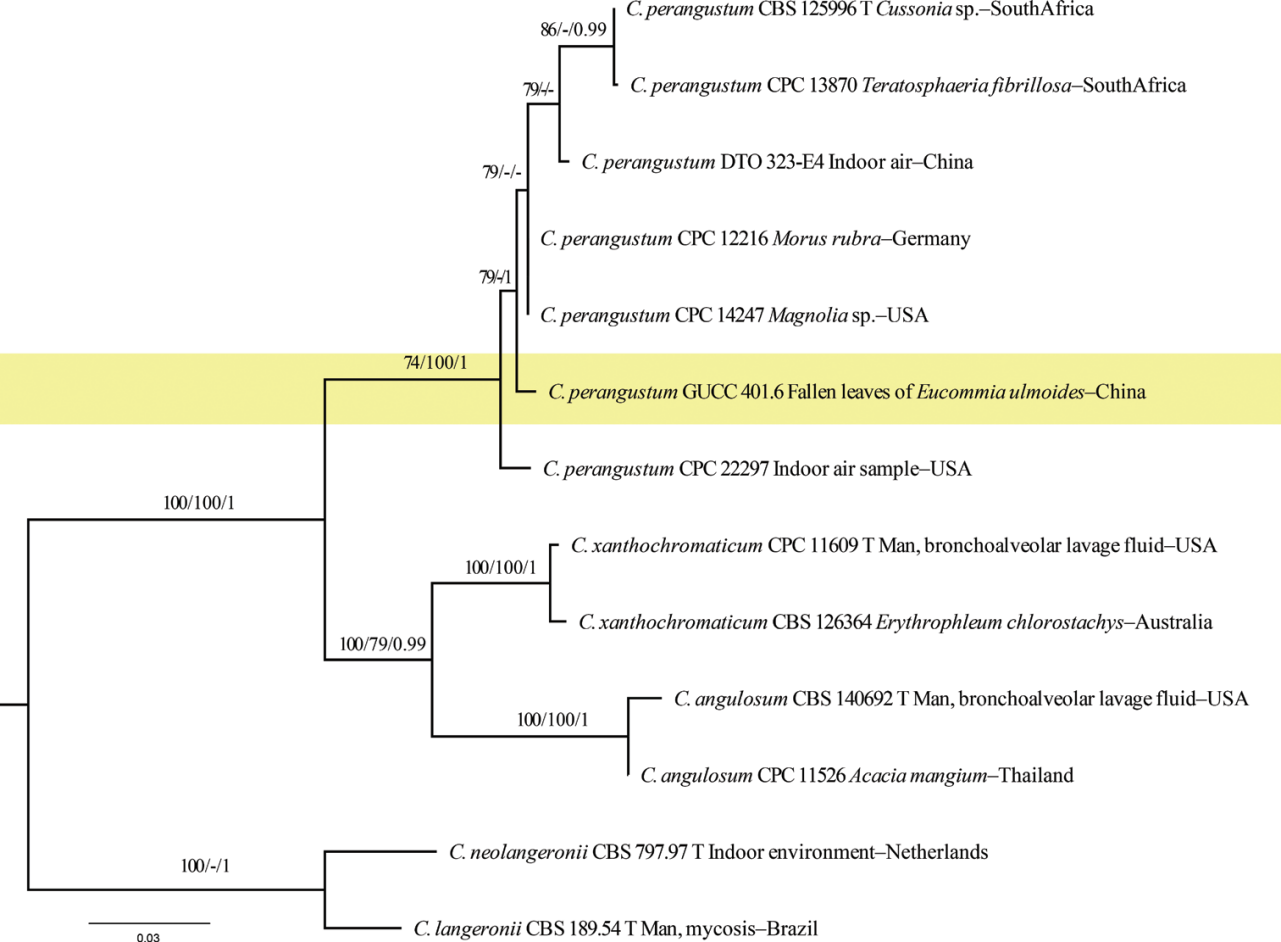
Maximum Likelihood (RAxML) tree from the combined analysis of ITS, *tef1* and *act* sequences of *Cladosporium*, which includes our strain GUCC 401.6. The tree was rooted with *C.neolangeronii* (CBS 797.97) and *C.langeronii* (CBS 189.54). ML and MP bootstrap values (≥ 70%) and Bayesian posterior probability (≥ 0.95) are indicated along branches (ML/MP/PP). Our species is highlighted with a yellow background. T = ex-holotype strain.

**Figure 2.**
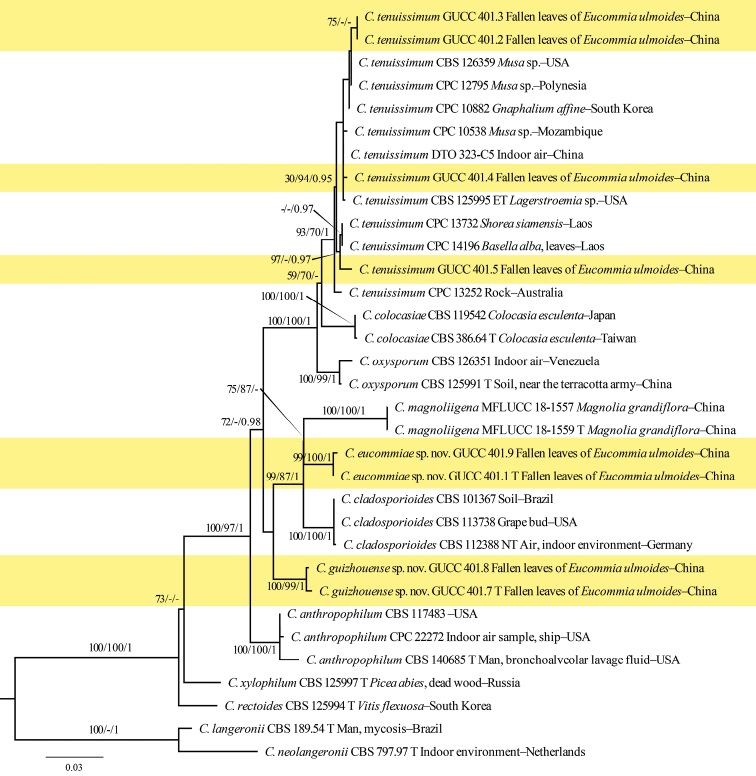
Maximum Likelihood (RAxML) tree from the combined analysis of ITS, *tef1* and *act* sequences of *Cladosporium*, which includes our strains GUCC 401.1–GUCC 401.9 (except for GUCC 401.6). The tree was rooted with *C.neolangeronii* (CBS 797.97) and *C.langeronii* (CBS 189.54). ML and MP bootstrap values (≥ 70%) and Bayesian posterior probability (≥ 0.95) are indicated along branches (ML/MP/PP). Our species are emphasized with a yellow background. T = ex-holotype strain, ET = ex-epitype strain, NT = ex-neotype strain.

DNA sequences used in this study (Table 1) were selected to obtain phylogenetic trees based on the closest matches by the BLASTn search with strain GUCC 401.6 and eight strains (GUCC 401.1–401.5 to GUCC 401.7–401.9), respectively, with outgroup *C.neolangeronii* (CBS 797.97) and *C.langeronii* (CBS 189.54). The final alignment (GUCC 401.6) of ITS, *tef1* and *act* comprised 1,033 characters, viz. ITS: 1–543, *act*: 544–770 and *tef1*: 771–1033, which included 843 constant characters, 38 variable characters and 152 parsimony-informative characters, and the alignment (GUCC 401.1–GUCC 401.9 except for GUCC 401.6) comprised 1,040 characters, viz., ITS: 1–544, *act*: 545–780 and *tef1*: 781–1040; which included 813 constant characters, 46 variable characters and 181 parsimony-informative characters. The RAxML results were selected to show the topology (Fig. 1 for GUCC 401.6 and Fig. 2 for GUCC 401.1–GUCC 401.9 except for GUCC 401.6), because the MP and Bayesian analyses resulted in similar topologies. The parameter settings that were used are shown in Table 3.

GUCC 401.6 clustered very close to *C.perangustum* (CBS 125996 = ex-holotype strain) with relatively high statistical support (79% MLBS/1 PP) (Fig. 1). Strains GUCC 401.2, GUCC 401.3, GUCC 401.4 and GUCC 401.5 had a very close relationship to *C.tenuissimum* (CBS 125995), variedly supported by MLBS (93%), PBS (70%) and PP (1) (Fig. 2). The comparison of DNA bases (Table 2) showed that our strains cluster with the ex-type strain of *C.tenuissimum* (CBS 125995, ex-epitype strain) with only one base pair difference in the ITS, two to fifteen base pair difference in the *tef1*, and one to five base pair difference in the *act. Cladosporiumeucommiae* (GUCC 401.1) is a sister to *C.magnoliigena* (MFLUCC 18-1559) and *C.cladosporioides* (CBS 101367) with high statistical support (75% MLBS / 87% MPBS)/(99 MLBS / 87% MPBS / 1 PP) (Fig. 2). The comparison of DNA bases composition (Table 2) indicated that, between *C.eucommiae* (GUCC 401.1) and *C.magnoliigena*, there were identical sequences in the ITS region, but 29 bases different in the *tef1* region. Unfortunately, *Cladosporiummagnoliigena* did not have *act* sequence data for comparison. The comparison of DNA bases composition (Table 2) indicated that, between *C.eucommiae* (GUCC 401.1) and *C.cladosporioides* (CBS 112388, ex-neotype strain), there were 18 bp differences in the *tef1* region, and 13 in the *act* region, but without difference in the ITS sequences. GUCC 401.7 was closer to *C.cladosporioides* (CBS 112388, ex-neotype strain) with high support in their respective branches (100% MLBS / 99% MPBS / 1 PP)/(100% MLBS / 100% MPBS / 1 PP) (Fig. 2). The comparison of DNA bases (Table 2) reveals 29 bp difference on *tef1* and 14 bp difference in *act* between *C.guizhouense* and *C.cladosporioides* (CBS 112388, ex-neotype strain), but only 1 bp difference in ITS sequences.

**Table 2.** The DNA base differences between our strains and related taxa in the three gene regions. Asterisks (*) denote our material.

Species	Strain number	Gene region and alignment positions		
		ITS (1–489 characters)	*tef1* (490–718 characters)	*act* (719–1008 characters)
*C.eucommiae* sp. nov.*	GUCC 401.1^T^	–	–	–
*C.eucommiae* sp. nov.*	GUCC 401.9	0	3	0
*C.magnoliigena*	MFLUCC 18-1559^T^	0	29	n/a
*C.magnoliigena*	MFLUCC 18-1557	0	29	n/a
*C.cladosporioides*	CBS 112388	0	18	13
*C.cladosporioides*	CBS 113738	0	16	13
*C.cladosporioides*	CBS 101367	1	16	13
		**ITS (1–542 characters)**	***tef1* (543–796 characters)**	***act* (797–1029 characters)**
*C.tenuissimum*	CBS 125995^ET^	–	–	–
C.tenuissimum*	GUCC 401.2	0	3	5
C.tenuissimum*	GUCC 401.3	0	15	n/a
C.tenuissimum*	GUCC 401.4	0	2	1
C.tenuissimum*	GUCC 401.5	1	9	3
		**ITS (1–507 characters)**	***tef1* (508–743 characters)**	***act* (744–948 characters)**
C.perangustum*	GUCC 401.6	–	–	–
*C.perangustum*	CBS 12599^6^T	0	26	7
*C.perangustum*	CPC 13870	0	22	7
*C.perangustum*	DTO 323-E4	0	13	5
*C.perangustum*	CPC 12216	0	2	5
*C.perangustum*	CPC 14247	0	2	5
		**ITS (1–480 characters)**	***tef1* (481–728 characters)**	***act* (729–933 characters**)
*C.guizhouense* sp. nov.*	GUCC 401.7^T^	–	–	–
*C.guizhouense* sp. nov.*	GUCC 401.8	0	3	2
*C.cladosporioides*	CBS 112388	1	29	14
*C.cladosporioides*	CBS 113738	1	27	14
*C.cladosporioides*	CBS 101367	2	27	14

**Table 3.** The parameters of MP and Bayesian methods in this study.

Strain number	MP						Bayesian		
	TL	PT	CI	RI	RC	HI	Model	Unique site patterns	ASDSF
GUCC 401.1 –GUCC 401.9 (except for GUCC 401.6)	400	300	0.7475	0.8648	0.6464	0.2525	ITS: JC+I; *tef1*: GTR+G; *act*: HKY+G	Division 1 = 54 Division 2 = 99 Division 3 = 154	0.009875
GUCC 401.6	281	2	0.8505	0.8817	0.7499	0.1495	ITS: SYM; *tef1*: GTR+G; *ac*t: GTR+G	Division 1 = 30 Division 2 = 75 Division 3 = 128	0.009961

TL: Tree length; PT: Parsimonious tree; CI: Consistency Indices; RI: Retention Indices; RC: Rescaled Consistency Indices HI: Homoplasy Index; Model: the models used for the different partitions; ASDSF: average standard deviation of split frequencies.

The pairwise homoplasy index (PHI) test revealed that there was no significant recombination (Фw = 0.4589) between *C.eucommiae* (GUCC 401.1 and GUCC 401.9) and the related taxa *C.magnoliigena*, *C.cladosporioides*, *C.guizhouense* (GUCC 401.7 and GUCC 401.8). The PHI test did not find any statistically significant evidence for recombination (Фw = 0.02487) between our four strains (GUCC 401.2, GUCC 401.3, GUCC 401.4 and GUCC 401.5) and the related taxon *C.tenuissimum* (CBS 126359, ex-epitype strain, CPC 12795, CPC 10882, CPC 10538, DTO 323-C5, CBS 125995, CPC 13732, CPC 14196 and CPC 13252). Based on the PHI test, there was a statistically significant recombination (Фw = 0.0104) between GUCC 401.6 and the related taxon *C.perangustum* (CBS 125996, = ex-holotype strain, CPC 13870, DTO 323-E4, CPC 12216, CPC 14247 and CPC 22297).

### ﻿Taxonomy

In this section, we introduced two new species and report two new substrate records.

#### ﻿ Cladosporium eucommiae

Taxon classificationFungiCladosporialesCladosporiaceae

S.Y. Wang, Yong Wang bis & Y. Li sp. nov.

0FD1C97D-4727-5963-8B7F-95861C669119

 842406

Fig. 3a–h

##### Etymology.

*eucommiae*, in reference to the genus name of the host plant (*Eucommiaulmoides*), from which the fungus was isolated.

**Figure 3.**
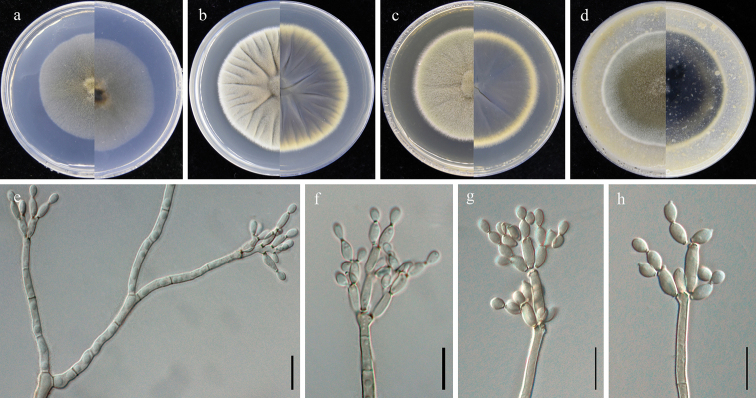
*Cladosporiumeucommiae* (GUCC 401.1, ex holotype strain). **a–d** colonies on SNA, PDA, MEA and OA (left: above, right: reverse) **e** branching conidiophore, secondary ramoconidia and conidia on SNA**f–h** conidiogenous cells, secondary ramoconidia and conidia on SNA. Scale bars: 10 µm (**e–h**).

##### Type.

China, Guizhou Province, Guiyang, Huaxi district, plantation forest of *E.ulmoides*, Guizhou University (26°24'16"N, 106°40'29"E), on fallen leaves of *E.ulmoides*, S.Y. Wang, Y. Wang & Y. Li, 13 January 2021 (HGUP 401.1, ***holotype***; ex-type living culture GUCC 401.1; additional living culture GUCC 401.9).

##### Description.

Saprobic on fallen leaves of *Eucommiaulmoides*. **Sexual morph**: Not developed. **Asexual morph**: Hyphomycetous. ***Mycelium*** abundant, superficially and submerged, overgrowing whole culture dishes, thin to dense, hyphae straight to slightly sinuous, branched, light olive-green to olive-brown, 1.5–5 µm wide, thin-walled, smooth. ***Conidiophores*** (7–)22–198 × 2.5–4.5 µm (*x̄*= 77.2 × 3.3 µm; n = 20), erect, branching, slightly attenuated towards the apex, light olive-green, smooth and thick-walled. ***Conidia*** 3–9 × 2.5–4.5 µm (*x̄*= 5.6 × 3.3 µm; n = 30), in simple and branched acropetal chains, mostly light olive, aseptate, smooth-walled and thin-walled, variable in size and shape, subglobose, ellipsoid-ovoid, obovoid, fusiform, subcylindrical. ***Secondary ramoconidia*** 5–25 × 2.5–4.0 µm (*x̄*= 11.9 × 3.4 µm; n = 30), olive-green, ellipsoid-ovoid, obovoid, fusiform, subcylindrical, aseptate, smooth-walled and thin-walled, rarely thick-walled.

##### Culture characteristics.

***Colonies*** on SNA 35–45 mm diam, after 2 weeks at 25 °C, pale olive, flat, velvety, with a regular edge, reverse light olive. ***Colonies*** on PDA 30–45 mm diam, after 2 weeks at 25 °C, olive-brown to gray-olive to iron-gray, with a regular white edge, irregularly folded, slightly depressed at the center, thatched, and often forming a bulge in the colony kernel, reverse olive to dark olive, with a whitish final edge. ***Colonies*** on MEA 35–45 mm diam, after 2 weeks at 25 °C, gray-green to olive, less radially furrowed, velvety, with an even gray white edge, reverse olive to dark olive, with an even gray-green final edge. ***Colonies*** on OA 35–40 mm diam, after 2 weeks at 25 °C, olive to gray-green, white at the final edge, flat, velvety, margin regular; reverse dark green to black, with a whitish final edge.

##### GenBank numbers.

ITS: OL587465, *tef1*: OL504966, *act*: OL519775 (GUCC 401.1); ITS: ON334729, *act*: ON383337 (GUCC 401.9).

#### ﻿ Cladosporium guizhouense

Taxon classificationFungiCladosporialesCladosporiaceae

S.Y. Wang, Yong Wang bis & Y. Li sp. nov.

8EBF93ED-34A6-56FE-9E6A-CC9FF9BC8BE3

 842407

Fig. 4a–h

##### Etymology.

guizhouense , in reference to the type location (Guizhou Province), where the fungus was isolated.

##### Type.

China, Guizhou Province, Guiyang, Huaxi district, plantation forest of *Eucommiaulmoides*, Guizhou University (26°24'16"N, 106°40'29"E), on fallen leaves of *E.ulmoides*, S.Y. Wang, Y. Wang & Y. Li, 13 January 2021 (HGUP 401.6, ***holotype***; living culture GUCC 401.7; additional living culture GUCC 401.8).

**Figure 4.**
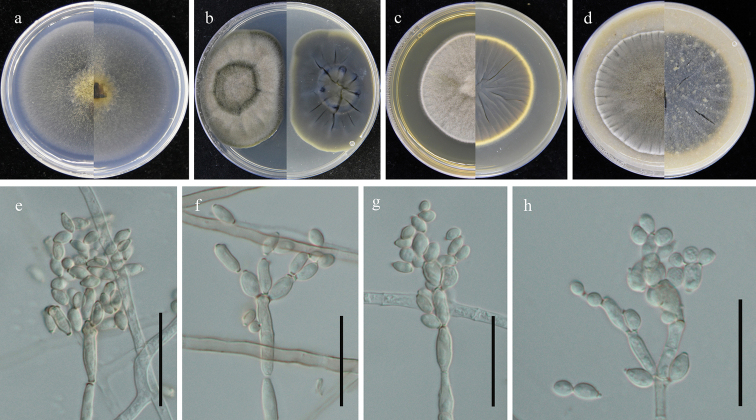
*Cladosporiumguizhouense* (GUCC 401.7). **a–d** colony on SNA, PDA, MEA and OA (left: above, right: reverse) **e–h** conidiogenous cells, secondary ramoconidia and conidia on SNA. Scale bars: 10 µm (**e–h**).

##### Description.

Saprobic on fallen leaves of *Eucommiaulmoides*. **Sexual morph**: Not developed. **Asexual morph**: Hyphomycetous. ***Mycelium*** abundant, submerged, overgrowing whole culture dishes, hyphae straight to slightly sinuous, septate, branching, light olive-green to olive-brown, mostly smooth- and thin-walled, 1.5–6 µm wide. ***Conidiophores*** 13–100 × 3–4.5 µm (*x̄*= 60.8 × 3.5 µm; n = 10), erect, branching, light olive-green, smooth- and thin-walled. ***Conidia*** 3–7.5 × 2.5–4 µm (*x̄*= 4.8 × 3.1 µm; n = 30), in simple and branched acropetal chains, mostly light olive, aseptate, mostly smooth- and thin-walled, variable in size and shape, ellipsoid-ovoid, obovoid, fusiform. ***Secondary ramoconidia*** 6.5–23 × 3–5.5 µm (*x̄*= 11.3 × 4.1 µm; n = 30), pale olive-green, narrowly ellipsoid to cylindrical-oblong, subcylindrical, aseptate, smooth- and thin-walled.

##### Culture characteristics:

***Colonies*** on SNA 45–55 mm diam, after 2 weeks at 25 °C, pale olive, flat, velvety, margin regularly, reverse light olive. ***Colonies*** on PDA 40–50 mm diam, after 2 weeks at 25 °C, smoke-gray to light olive-gray, reverse leaden-gray, gray-olive at edge both surface and reverse, woolly or felty, broad edge, regular, growth low convex, without protuberant exudates, reverse formed cracks in the middle small circle. ***Colonies*** on MEA 30–40 mm diam, after 2 weeks at 25 °C, smoke-gray to light olive-gray, woolly or felty, fluffy, with a whitish narrow final edge; reverse olive-yellow or olive-brown, radially furrowed, irregularly folded, with a whitish narrow final edge. ***Colonies*** on OA 30–45 mm diam, after 2 weeks at 25 °C, gray-green or olive, granular and fluffy mycelium, woolly and felty edge, with an irregularly folded whitish and olive final edge; reverse olive-yellow or olive-brown, with a whitish narrow final edge.

##### GenBank numbers.

ITS: OL579741, *tef1*: OL504965, *act*: OL519780 (GUCC 401.7); ITS: ON334728, *tef1*: ON383470, *act*: ON383338 (GUCC 401.8).

#### ﻿ Cladosporium perangustum

Taxon classificationFungiCladosporialesCladosporiaceae

Bensch, Crous & U. Braun, Studies in Mycology 67: 65 (2010)

73121E75-2E1E-5644-BC97-A4D9ABDAC581

 517085

Fig. 5a–h

##### Material examined.

China, Guizhou Province, Guiyang, Huaxi district, plantation forest of *E.ulmoides*, Guizhou University (26°24'16"N, 106°40'29"E), on fallen leaves of *E.ulmoides*, S.Y. Wang, Y. Wang & Y. Li, 13 January 2021 (HGUP 401.6, living culture GUCC 401.6) (new substrate record).

**Figure 5.**
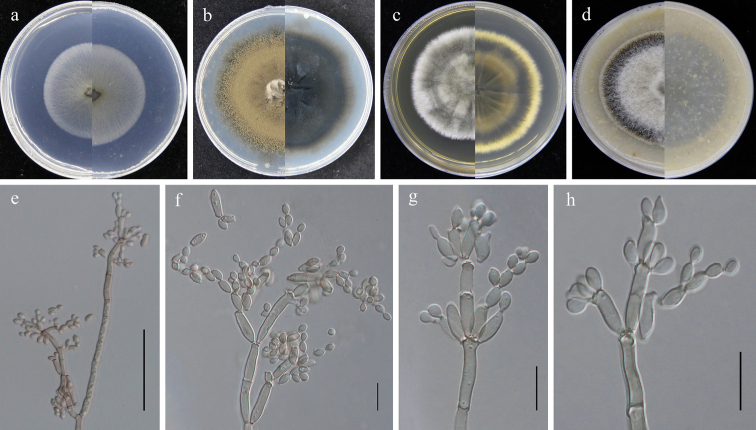
*Cladosporiumperangustum* (GUCC 401.6, new substrate record from Guizhou Province). **a–d** colonies on SNA, PDA, MEA and OA (left: above, right: reverse) **e** branching conidiophore, secondary ramoconidia and conidia on SNA**f–h** conidiogenous cells, secondary ramoconidia and conidia on SNA. Scale bars: 50 µm (**e**); 10 µm (**f–h**).

##### Description.

Saprobic on fallen leaves of *Eucommiaulmoides*. **Sexual morph**: Not developed. **Asexual morph**: Hyphomycetous. ***Mycelium*** superficial, hyphae branched, hyaline to subhyaline, 2.5–5 μm wide, usually slightly constricted at the septa and somewhat swollen, smooth to somewhat verruculose or irregularly rough-walled. ***Conidiophores*** macro- and micronematous, 14–167 × 2.5–4.5 µm (*x̄*= 65.4 × 3.4 µm; n = 20), erect, branched, slightly attenuated towards the apex, light olive-green, smooth and thick-walled. ***Conidia*** in acropetal chains, 2–9.5 × 2–4 µm (*x̄*= 5.6 × 3.3 µm; n = 30) mostly light olive-green, aseptate, mostly smooth-walled and thin-walled, variable in size and shape, subglobose, ellipsoid-ovoid, obovoid, fusiform. ***Secondary ramoconidia*** 6–24 × 2–5.5 µm (*x̄*= 11.2 × 3.3 µm; n = 30), olive-green, narrowly ellipsoid to cylindrical-oblong, subcylindrical, aseptate, rarely 1-septate, mostly smooth-walled and thick-walled.

##### Culture characteristics.

***Colonies*** on SNA 30–40 mm diam, after 2 weeks at 25 °C, pale olive to pale whitish, flat, velvety, with a regular edge, reverse light olive to light white. ***Colonies*** on PDA 30–40 mm diam, after 2 weeks at 25 °C, olive-gray to olive-green or olive-brown, powdery or flocculent, fluffy, regular, radially furrowed, lacerated or feathery, and often forming a gray-white or olive bulge in the colony kernel; reverse dark olive or dull green to black. ***Colonies*** on MEA 35–45 mm diam, after 2 weeks at 25 °C, gray-green to white or gray-white, fluffy, radially furrowed, with a whitish final edge; reverse olive-yellow to olive-gray to olive-green, with a whitish final edge. ***Colonies*** on OA 35–45 mm diam, after 2 weeks at 25°C, white to olive-green, with a pale gray final edge, velvety or fluffy, margins colorless or pale gray, glabrous, regular; reverse olive-green to dark green.

##### GenBank numbers.

ITS: OL579742, *tef1*: OL581726, *act*: OL519779.

#### ﻿ Cladosporium tenuissimum

Taxon classificationFungiCladosporialesCladosporiaceae

Cooke, Grevillea 6: 140 (1878)

A62A7F1E-3CA4-5C0B-A9FB-E5D431BEF725

 145672

Fig. 6a–i

##### Materials examined.

China, Guizhou Province, Guiyang, Huaxi district, plantation forest of *E.ulmoides*, Guizhou University (26°24'16"N, 106°40'29"E), on fallen leaves of *E.ulmoides*, S.Y. Wang, Y. Wang & Y. Li, 13 January 2021, (HGUP 401.1; HGUP 401.2; HGUP 401.3 and HGUP 401.4, living cultures GUCC 401.2; GUCC 401.3; GUCC 401.4 and GUCC 401.5) (new substrate record).

**Figure 6.**
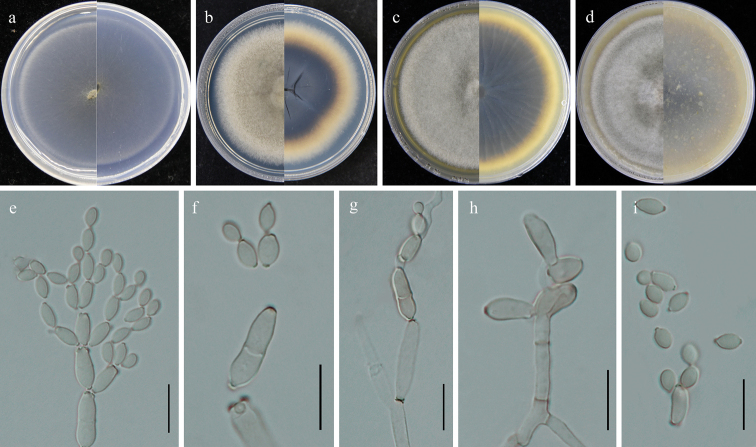
*Cladosporiumtenuissimum* (GUCC 401.2, new substrate record from Guizhou Province). **a–d** colonies on SNA, PDA, MEA and OA (left: above, right: reverse) **e–h** secondary ramoconidia and conidia on SNA**i** conidia on SNA. Scale bars: 10 µm (**e–i**).

##### Description.

Saprobic on fallen leaves of *Eucommiaulmoides*. **Sexual morph**: Not developed. **Asexual morph**: Hyphomycetous. ***Mycelium*** abundant, superficial and submerged, overgrowing whole culture dishes, hyphae straight to slightly sinuous, septate, branching, light olive-green to olive-brown, smooth-walled, 1.5–6 µm wide. ***Conidiophores*** 13–100 × 2.5–4.5 µm (*x̄*= 60.8 × 3.6 µm; n = 10), erect, branching, light olive-green, smooth and thin walled. ***Conidia*** 2.5–7.5 × 2–4 µm (*x̄*= 4.9 × 3.2 µm; n = 30), in simple and branched acropetal chains, mostly light olive-green, aseptate, mostly smooth- and thin-walled, variable in size and shape, subglobose, ellipsoid-ovoid, obovoid, fusiform. ***Secondary ramoconidia*** 5.5–23 × 2.5–5.5 µm (*x̄*= 0.9 × 3.8 µm; n = 30), pale olive-green, narrowly ellipsoid to cylindrical-oblong or subcylindrical, sometimes septate and sometimes aspetate (1-septate appear at maturity), smooth- and thin-walled.

##### Culture characteristics:

***Colonies*** on SNA 50–55 mm diam, after 2 weeks at 25 °C, pale olive to pale white, flat, velvety, with a regular edge, reverse light olive to light white. ***Colonies*** on PDA 40–55 mm diam, after 2 weeks at 25 °C, smoke-gray to light olive-gray or olive to light olive-gray, reverse leaden-gray, gray-olive at edge both surface and reverse, woolly or felty, broad edge, regular, growth low convex, without protuberant exudates, occasionally reverse formed a sunflower like shape in the middle. ***Colonies*** on MEA 40–50 mm diam, after 2 weeks at 25 °C, olive-gray or gray, fluffy; reverse olive-green to dark olive, with an olive-yellow to gray-white edge, radially furrowed. ***Colonies*** on OA 40–60 mm diam, after 2 weeks at 25 °C, gray-white or iron-gray to gray-olive, fluffy to felty; reverse olive-brown to olive.

##### GenBank numbers.

ITS: OL579746, *tef1*: OL504967, *act*: OL519776 (GUCC 401.2); ITS: OL579745, *tef1*: OL505077 (GUCC 401.3); ITS: OL579744, *tef1*: OL581724, *act*: OL519777 (GUCC 401.4); ITS: OL579743, *tef1*: OL581725, *act*: OL519778 (GUCC 401.5).

## ﻿Discussion

In this paper, we revealed four *Cladosporium* taxa on fallen leaves of *E.ulmoides*, two of which are described here as new to science. Phylogenetic analyses showed that *C.eucommiae* is different from *C.magnoliigena* (Jayasiri et al. 2019), although *act* sequences are not available for the latter species. Conidia of *C.eucommiae* (3–9 × 2.5–4.5 µm) are usually narrower and longer than those of *C.magnoliigena* (4.2–5.5 × 2–5 µm), while secondary ramoconidia of *C.eucommiae* are usually aseptate and longer than those of *C.magnoliigena* (5–25 × 2.5–4.0 µm *vs* 9.5–18 × 2.7–4.2 µm and 0–3-septate). Thus, the two species are clearly distinct in morphology as well as DNA sequence data. Phylogenetic analyses showed that sequences retrieved from GUCC 401.7 and GUCC 401.8 are different from those obtained from *C.cladosporioides* (CBS 112388, ex-neotype strain) (Bensch et al. 2010) by phylogenetic analyses (Fig. 2). Conidia of GUCC 401.7 and *C.cladosporioides* show no significant differences in size, color and shape, but secondary ramoconidia of GUCC 401.7 were usually shorter than those of *C.cladosporioides* (6.5–23 × 3–5.5 µm *vs* 15–50 × (2.5–)3–5 µm), and conidiophores of GUCC 401.7 (13–100 × 3–4.5 µm) were shorter than in *C.cladosporioides* (40–300(–350) × (2.5–)3–4(–5.5) µm). Therefore, there are significant differences in the morphology and DNA sequence data between the two species. The combination of morphology, phylogenetic analyses, comparison of DNA base composition and GCPSR analysis support our proposal that *C.eucommiae* and *C.guizhouense* represent two novel taxa.

Sequences retrieved from GUCC 401.6 clustered among six sequences obtained from *C.perangustum* strains (Fig. 1), but conidia of GUCC 401.6 (2–9.5 × 2–4 µm) were usually somewhat narrower and longer than CBS 125996 (Bensch et al. 2010) (2–4(–5) × (1.5–)2–2.5 µm), and secondary ramoconidia of GUCC 401.6 (6–24 × 2–5.5 µm) were wider than those of *C.perangustum* (6–30(–34) × 2–3(–3.5) µm). In addition, GUCC 401.6 can be well distinguished from *C.perangustum* by its slower growing colonies in PDA, MEA and OA (30–40, 35–45 and 35–45 mm diam/14 d), whereas CBS 125996 grew 33–76, 40–72 and 40–75 mm diam/14 d. Although morphology and phylogeny showed minor differences, GCPSR analysis supported statistically significant recombination, after careful consideration, GUCC 401.6 was identified as *C.perangustum*. The differences may be caused by different substrates or geographical regions, which needs further investigation. Conidiophores of GUCC 401.2–GUCC 401.5 were shorter than in CBS 125995 (13–100 × 2.5–4.5 µm *vs* 49–542(–800) × (3–)4–7 µm), but secondary ramoconidia and conidia (5.5–23 × 2.5–5.5 µm; 2.5–7.5 × 2–4 µm) were similar to those of *C.tenuissimum* (15–31 × 4–5 µm; 3–13 × 2–6 µm) (Cooke 1878). Sequences retrieved from our four strains cluster with sequences obtained from *C.tenuissimum* strains (Fig. 2) with minor DNA base differences. Thus, our four strains were identified as *C.tenuissimum*.

Our five strains pertain to two known species, viz., *C.perangustum* and *C.tenuissimum*, but with *E.ulmoides* as new substrate records for these species. The main focus of this study was the exploration of the diversity of microfungi associated with a *E.ulmoides* plantation forest. In previous studies, *Cladosporiumparapenidielloides* was found on *Eucalyptus* sp. in Australia, *C.perangustum* on *Magnolia* sp. in the USA, and *C.pini-ponderosae* on *Pinusponderosa* in Argentina. So far, *Cladosporium* species have never been isolated from fallen leaves of *E.ulmoides*, the only species of the genus *Eucommia*.

## Supplementary Material

XML Treatment for Cladosporium eucommiae

XML Treatment for Cladosporium guizhouense

XML Treatment for Cladosporium perangustum

XML Treatment for Cladosporium tenuissimum
